# Assessing the Psychological Priorities for Optimising Captive Asian Elephant (*Elephas maximus*) Welfare

**DOI:** 10.3390/ani10010039

**Published:** 2019-12-23

**Authors:** Jake Stuart Veasey

**Affiliations:** School of Animal, Rural and Environmental Sciences, Nottingham Trent University, Southwell NG25 0QF, UK; jake@carefortherare.com

**Keywords:** animal welfare, appetitive, Asian elephant, AWPIS^©^, behavioural needs, cognition, motivation, psychological priorities, zoo

## Abstract

**Simple Summary:**

The welfare of elephants in captivity is of significant public interest and the cause of considerable debate amongst the scientific, legislative, zoo and animal welfare advocacy communities. A tool capable of identifying what elephants need to experience to have good welfare would not only help bring clarity to this debate, it could also direct elephant welfare policy and management to more effectively optimise welfare and provide a valuable reference tool by which elephant welfare could be assessed. To that end, a systematic process is trialed to identify the welfare priorities for Asian elephants. These pilot assessments demonstrate the importance of providing species-appropriate feeding, social and mental opportunities to protect elephant welfare and suggest that the current priorities established in husbandry guidelines do not accurately reflect the psychological needs of elephants; in particular, they appear to underestimate the importance of behaviours and mental processes associated with acquiring food.

**Abstract:**

The welfare status of elephants under human care has been a contentious issue for two decades or more in numerous western countries. Much effort has gone into assessing the welfare of captive elephants at individual and population levels with little consensus having been achieved in relation to both the welfare requirements of captive elephants, or their absolute welfare status. A methodology capable of identifying the psychological priorities of elephants would greatly assist in both managing and assessing captive elephant welfare. Here, a Delphi-based Animal Welfare Priority Identification System^©^ (APWIS^©^) is trialled to evaluate the reliability of the methodology and to determine the welfare significance of individual behaviours and cognitive processes for Asian elephants (*Elaphus maximus*). APWIS^©^ examines the motivational characteristics, evolutionary significance and established welfare impacts of individual behaviours and cognitive processes of each species being assessed. The assessment carried out here indicates appetitive behaviours essential for survival in the wild, together species-specific social and cognitive opportunities are likely to be important to the welfare of Asian elephant in captivity. The output of this assessment, for the first time, provides comprehensive species-specific psychological/welfare priorities for Asian elephants that should be used to inform husbandry guidelines, habitat design and management strategies and can also provide a valuable reference tool for Asian elephant welfare assessment. The effective application of these insights could lead to substantive improvements in captive Asian elephant welfare.

## 1. Introduction

Whether captive elephants have adequate welfare is a contentious issue [1,2,3,4,5]. Some stakeholders argue that thwarted natural behaviour patterns, a lack of choice, control and freedom together with persistent health problems all compromise the welfare of captive elephants [5,6]. However, such claims are typically refuted by those responsible for captive elephant management. Much effort has gone into assessing the welfare status of individual captive elephants as well as across populations [1,2,5,6,7,8,9,10,11,12,13,14], however, comparatively little has been written on identifying how to actively provision optimal welfare states for elephants. 

Analysis of welfare assessments can provide insights into likely welfare impacts of management priorities and habitat design, but such an approach is inevitably constrained by the challenges inherent in the nature of welfare assessments, see [4,6,9,10,15,16], as well as the nature of the pre-existing management conditions in which elephants are maintained. The capacity of population-wide welfare assessments such as those undertaken by Meehan et al. [2], Clubb and Mason [5] and Mason and Veasey [6] to identify conditions necessary to deliver optimal elephant welfare are inevitably constrained by the quality of habitats assessed, which may all be qualitively or quantitively deficient in features necessary to guarantee optimal welfare. 

Williams et al. [1] reviewed existing elephant welfare assessments and concluded that of thirty-seven criteria used in thirty peer reviewed papers; stereotypies, glucocorticoids and body condition were likely to be the most reliable welfare indicators. Mason and Veasey [9,10] attempted to identify likely mechanisms by which elephant welfare might be examined and compared data relating to reproductive success, mortality and stereotypic behaviours for zoo elephants with benchmark populations in range states. Their analysis suggested that on a population level, zoo elephants at that time, were likely to experience poorer welfare than benchmark populations in range states [6]. However informative this research may have been regarding population level captive elephant welfare at that time, it did not identify welfare priorities, establish the welfare status of individual elephants and nor did it provide clear guidance on how elephant welfare might be improved. Similarly, whilst Williams et al. [1] identified walking as a popular criterion for assessing welfare, they acknowledged that little is known about how far elephants should walk in order to experience good welfare. Management [2] and sociality [2,14] have both been identified as being factors likely to influence captive elephant welfare but neither study was able to provide guidance on what optimal management or the ideal social circumstances might be for elephant welfare. Veasey [4] attempted to provide some suggestions as to what might be important to elephant welfare, but at that time, and until now, there has been no systematic evidence-based process to identify psychological priorities for captive elephants. 

Before attempting to identify the psychological priorities necessary for optimal captive Asian elephant (*Elephas maximus*) welfare, it is essential to establish a clear understanding of what is meant by animal welfare. For the purposes of this assessment, Mason and Veasey’s conception of animal welfare has been used [6,9,10] which considers poor welfare to occur when animals experience severe or chronic states of mental suffering and good welfare to occur when animals experience positive emotional states and negligible mental suffering. 

Whilst it is evident that the physical health of an animal can impact its welfare and vice versa, physical wellbeing is not directly referenced in this and other feelings-based conceptions of animal welfare because physical health is only relevant to welfare if it affects how animals feel, see [6,9,10,15,16,17,18,19,20,21,22,23]. Moreover, this assessment is intended to identify the psychological priorities of Asian elephants, not their physical needs which are arguably more widely understood and comprehensively addressed in existing husbandry guidelines [24,25]. The output from the Animal Welfare Priority Identification System^©^ (AWPIS^©^) process described here is thus intended to complement the functional components of welfare relating to nutrition, environment and health covered in existing frameworks and husbandry guidelines, see [24,25,26,27,28] rather than simply replacing them.

However, because the feelings of animals including elephants remain largely closed to us, there are considerable challenges in both assessing and prioritising captive care [4,15,16,21,29,30,31,32], which frequently results in both animal welfare assessment and animal welfare management focusing unduly on more tangible metrics linked to physical health [15,18,22]. Veasey [15] argues that management that prioritizes the physical wellbeing of animals including captive Asian elephants, often does so at the expense of the psychological needs of those animals, reflecting an inherent conflict between physical and psychological priorities in relation to captive animal welfare management.

The extent to which captive Asian elephants can be protected from physical harms is governed by the capacity of carers to provide for the essential individual physical needs of elephants, mitigate naturally occurring and anthropogenic risk factors routinely experienced in nature and in captivity, whilst maintaining the capacity to observe and intervene. For captive elephants, this is characterised close behavioural monitoring, extensive preventative health care programs typically including routine foot examinations, blood sampling and TB surveillance as well as the capacity intervene when necessary. In contrast, protecting the psychological needs of captive elephants is likely to require the provisioning of appropriate ‘freedoms’ in the form of behavioural and cognitive opportunities appropriate to the species and individual. These might include the freedom to roam, to forage, to breed, to fight, to seek seclusion or the company of others, to successfully respond to challenges, to take risks and to make choices which might include choosing not participating in husbandry activities. For large, social, tropical and subtropical animals as Asian elephants, not only may these freedoms be challenging to provide for in captivity, they may also conflict directly with many of the conditions routinely considered desirable for safeguarding physical wellbeing [15]. 

For example, foot health has long been an issue of considerable concern for captive Asian elephants [8,33,34,35,36] and in response to such concerns, the Association of Zoos and Aquariums (AZA) Standards of Elephant Management and Care recommended, amongst other steps, routine footcare be provided including baseline radiographs together with exercise programs [24,35]. Whilst this strategy was reportedly effective in reducing the incidence of foot pathologies [35], it is unclear what if any impact the management necessary to meet these requirements has had on the psychological wellbeing of elephants, and thus its overall impact on welfare. 

For many years, it had been widely accepted that free contact management where staff enter the same unprotected space as the elephants, was the most effective management system with which to provide footcare. This was in spite of the risks to keepers and the potentially negative impacts on the psychological wellbeing of elephants including fear and pain resulting from the use of tools such as the ankus or bull-hook, the separation of tight knit social units for free contact training and management purposes and the reduction in time available for elephants to behave as they would choose to. In more recent years, however, free contact management has fallen out of favour, largely due to concerns over human safety. As of 2016, within AZA zoos, staff interact directly with Asian elephants for an average of 56.9% of zoo operating hours [11], reflecting a growing emphasis on training as a primary management tool to maintain wellbeing and foot health [11,35,36]. Veasey challenged such intensive management and treatment-based approaches, advocating instead for a greater emphasis on environmental and behavioural ecological solutions to the physical and psychological challenges elephants face in captivity [4]. This thinking was incorporated into the BIAZA elephant husbandry guidelines, which rather than mandating footcare, mandate zoos develop habitats in which foot problems are unlikely to occur [25], which has proven to be an important step in shifting elephant management practices over the past two decades. However, despite the ongoing evolution in elephant management, thus far, there has yet to be a systematic attempt to identify what the psychological priorities of Asian elephants are in order to better balance physical and psychological needs of the species, understand the impact of training and management on the welfare of elephants, and ultimately to further improve captive elephant welfare.

The aim of this investigation is two-fold; firstly, to explore the viability of AWPIS^©^ to evaluate the psychological priorities of a species using the Asian elephant as a model species, and secondly, to better understand the psychological priorities of Asian elephants in order to optimise their welfare in captivity.

## 2. Materials and Methods

The assessment outlined here for Asian elephants utilises the proprietary Animal Welfare Priority Identification System^©^ (AWPIS^©^) described by Veasey [37] that systematically quantifies the psychological significance of behaviours and cognitive processes for animals such that welfare centric priorities can be established. 

AWPIS^©^ is loosely based around the Delphi process; a methodology originally developed to obtain consensus from a panel of experts tasked with predicting scenario-based outcomes using questionnaires and feedback [38] and relying on the collective wisdom of an appropriately qualified group rather than a single expert [39].

Panellists are not asked to provide subjective opinions on the importance of behaviours and cognitive processes to Asian elephant welfare but are instead tasked with ranking each behaviour and cognitive process from one to five according to twelve criteria, each with their own objective ranking formula. Collectively, these twelve criteria reflect the evolutionary significance of each behaviour and cognitive process, provide insights into their motivational characteristics and evaluates evidence of known welfare impacts for their expression or non-expression where that has been demonstrated [37].

The methodology is premised on the fact that motivation is an evolved mechanism to elicit behaviours and cognitive processes to solve evolutionary important challenges [40,41,42,43,44,45,46,47,48,49,50]. Thus, behaviours or cognitive processes of high evolutionary significance will be similarly highly motivated for when stimulated. Furthermore, the extent to which an animal suffers as a result of being frustrated in its desire to express a behaviour or cognitive process will be broadly proportional to the strength of the frustrated motivation. It subsequently follows that a relationship exists between the evolutionary significance of a behaviour and its relevance to animal welfare; if animals are frustrated in their desire to express highly motivated naturally occurring behaviours or cognitive processes, the welfare impact will be broadly proportional to their evolutionary significance, see [15,51]. 

Accordingly, seven of the twelve assessment criteria seek to evaluate the evolutionary significance of each behaviour and cognitive process by referencing the behavioural ecology of the species in its natural state. Three further criteria consider the motivational characteristics of each behaviour and cognitive process including the strength and frequency of their motivation as well as the origin of the motivating stimulus. Understanding the origin of the stimulus ensures the process is able to distinguish between behaviours and cognitive processes of high evolutionary significance that are exclusively or predominantly triggered by external factors, which may never become manifest in captive environments. The non-expression of behaviours or cognitive processes which may never be stimulated in captivity such as prey species evading predators, cannot be assumed to compromise welfare without further supporting evidence, see [15,52,53]. The remaining two criteria consider the likely welfare impacts of Asian elephants being able, or not, to express behaviours and cognitive processes where they have been assessed, and is based primarily on published research on Asian elephant welfare derived from captive studies. 

Whilst AWPIS^©^ assessments take place in the form of facilitated group events, panellists independently rank each behaviour or cognitive processes against each of the twelve criteria using an online assessment platform, with clear ranking guidelines and the support of a trained facilitator. The platform utilises a proprietary algorithm to collate the ranks provided by the panellists, for each of the twelve criteria, applied against each behaviour and cognitive process into a single AWPIS^©^ score which reflects the collective feedback of the panel for each behaviour and cognitive process.

By reviewing the input from the panel on the online platform in real time, the facilitator is able to identify anomalies that might emerge where there is a disparity in ranking from within the panel exceeding three from a possible range of five. At such a juncture, the facilitator reviews the definition of the specific behaviour or cognitive process being considered with the panel as well as the assessment criteria it is being ranked against, together with the guidelines for ranking to ensure there is universal understanding. Differences of opinion are acceptable, but it is the role of the facilitator to avoid differences in understanding of the assessment criteria, the ranking framework or the behaviour and cognitive processes being considered. In reality, such outliers have proven to be extremely rare. Moreover, because AWPIS^©^ is configured to utilise 12 criteria and panels typically comprised of 10–20 members, unresolved irregularities in ranking from within the panel would have limited impact upon the overall AWPIS^©^ rank for each behaviour and cognitive process.

For the purpose of this assessment, panellists were asked to assess the needs of Asian elephants as a species, including both sexes and all age classes rather than considering specific cohorts. Were an understanding of the needs of a particular sex or age class required, the assessment would have specifically targeted the needs of that cohort. Thus, the importance of specific behaviours or cognitive processes such as nursing, play and learning in a lifetime species-based assessment such as this will not accurately reflect the needs of specific cohorts such as juveniles or nursing mothers, which needs to be considered in the interpretation of the output. It should also be noted that although Asian elephants have been in captivity for millennia, they are not considered to be a domesticated species [54] because recruitment has predominately been from wild populations. So, whilst captive behaviour is considered in the AWPIS^©^ process, assessments emphasise the importance of the species’ behavioural ecology in the natural state rather than focussing primarily on captive behaviours and adaptive responses made by individual elephants to the captive state.

In order to validate the AWPIS^©^ tool in being able to consistently evaluate the psychological significance of behaviours and cognitive processes, and to gain a better understanding of the psychological needs of the Asian elephant in captivity, two assessments were undertaken opportunistically as a component of two separate workshops; one considering elephant welfare issues in Vietnam and the other as part of a zoo animal welfare training workshop. The first assessment was carried out by a panel convened to explore welfare challenges associated with elephants used in tourism in Vietnam and is subsequently referred to as the ‘in-situ’ assessment. Eight panellists successfully completed this assessment comprised of animal welfare specialists and in-situ and captive management experts. The second assessment was convened in Sweden and carried out exclusively by 26 zoo professionals studying on the European Association of Zoo’s and Aquarium’s (EAZA) Advanced Animal Welfare Course and is subsequently referred to as the ‘ex-situ’ assessment. These experts were all well versed in captive elephant management and biology but had no experience of the species in-situ, and little or no prior formal training in animal welfare science.

## 3. Results

**Figure 1:**
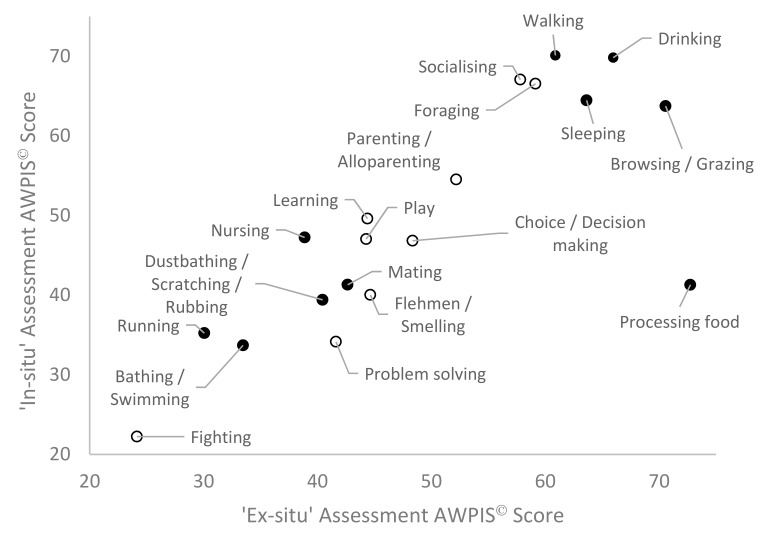
Correlation of AWPIS^©^ scores for Asian elephant behaviours and cognitive processes provided by two independent assessments; Pearson’s correlation *n* = 19, r = 0.789, *p* ≤ 0.001. Open circles denote cognitive process or behaviours with significant cognitive components to them.

Despite the differing makeup of the panels, there was a strong correlation between the AWPIS^©^ scores provided by the two panels for each of the behaviours and cognitive processes for Asian elephants (see Figure 1, Pearson’s correlation, *n* = 19, r = 0.789, *p* ≤ 0.001).

**Figure 2:**
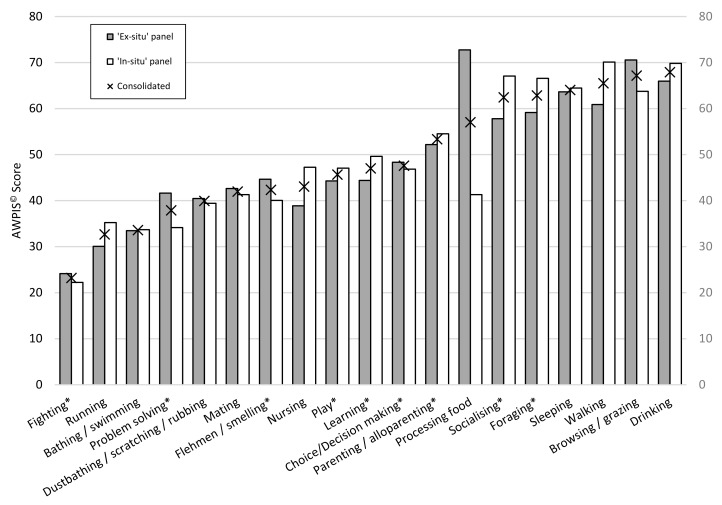
AWPIS^©^ scores for each behaviour and cognitive process for ‘ex-situ’ assessments (grey bars) and ‘in-situ’ assessments (open bars), together with data consolidated from both ‘in-situ’ and ‘ex-situ’ assessments (marked with an X) by which behaviours and cognitive processes are ultimately ranked. * Denotes cognitive process or behaviours with significant cognitive components to them.

Behaviours essential for the maintenance of physical health were unsurprisingly identified as being amongst the most important welfare priorities by both assessment panels. Despite the strong correlation between the AWPIS^©^ scores produced by the two panels, there were also noteworthy differences (see Figure 2). The biggest discrepancy between the ex-situ and in-situ panels was in the ranking of processing food with the ex-situ professionals ranking it as the greatest priority with an AWPIS^©^ score of 72.7 compared to the in-situ experts scoring it 41.3. 

The next greatest discrepancies were for socialising, walking and nursing with the in-situ panel scoring their importance higher than the ex-situ panel, followed by problem solving which was ranked higher by the ex-situ panel. However, directly comparing the AWPIS^©^ scores for each behaviour and cognitive process between the two panels, there was a ±11.5% average deviation between the two panels. The removal of largest outlier (processing food) improved the strength of the already significant correlation markedly (*n* = 18, r = 0.935, *p* ≤ 0.001). Regardless of this particular discrepancy between the two panels, the strong correlation between the two assessments justifies their consolidation into a single AWPIS^©^ score for each behaviour and cognitive process. 

The AWPIS^©^ scores set out in Figure 2 place behaviours and cognitive processes in the order likely to represent their relative significance to the welfare of Asian elephants in captivity across a population, including both sexes and all ages based on the consolidated data, ranging from high to low, from right to left. 

## 4. Discussion

The purpose of this assessment was two-fold; firstly, to determine whether the AWPIS^©^ methodology is a consistent process with which to identify welfare priorities and secondly to provide an insight into the welfare priorities for captive Asian elephants. 

The strong correlation between the two assessments despite markedly differing characteristics between the two panels, as well as minor iterative adjustments in the delivery of the process demonstrates the effectiveness of this methodology in establishing a defendable, repeatable framework with which to identify the psychological priorities of a species. Such insights are invaluable to the optimisation of animal management and habitat design and has, up and till now been lacking, see also [37]. Whilst the necessity to carry out two independent assessments with differing panel configurations to determine the repeatability of the process requires a degree of caution being applied to the current output, it nonetheless provides a unique insight into the likely psychological priorities for captive Asian elephants.

Whilst the discrepancies between the two assessments are not sufficiently great for the data not to be combined, they do appear to reflect the differing backgrounds of the two panels, supporting the premise that assessments should include a broad, balanced constituency of relevant experts [37]. Panels should ideally include experts with insights from the species in the wild who will have essential knowledge relating to natural behaviours in an evolutionary appropriate context as well as those with experience of the species in captivity, who due to their intimate relationship with individuals of the species, will have access to unprecedented insights into motivations, preferences and impacts of specific deprivations and opportunities on welfare. Finally, the assessment should include panellists with insights into the welfare of elephants in captivity from a scientific perspective.

In this instance, the ex-situ panel appear to have elevated the importance of problem solving and food processing, likely reflecting management techniques that are widely used in the behavioural enrichment of captive elephants to make up for the reduced amount of time spent foraging in captivity. Interestingly, the ex-situ panel, also ranked browsing/grazing as higher than the in-situ panel, also potentially reflecting the reduced amount of time foraging in captivity. In contrast, foraging, walking, socialising and nursing were ranked higher by the in-situ panel, likely reflecting the differences in behaviour and social circumstances between wild and captive elephants (see Figure 2). 

Whilst it was not the purpose of this paper to comprehensively consider the application of the findings of the assessment to captive Asian elephant management, there are a number of notable findings worthy of consideration. 

The consolidated AWPIS^©^ assessment for Asian elephants ranked drinking and browsing/grazing as the most important behaviours from the ethogram of Asian elephants used in this assessment. However, it should not be interpreted that satisfying the physical needs of Asian elephants associated with feeding and drinking, would also satisfy the psychological priorities associated with those behaviours, see [15,43]. So, for example, whilst providing browse might satisfy specific nutritional requirements of Asian elephants, it is unlikely to satisfy all the behavioural or cognitive requirements associated with browsing such as selecting, harvesting and processing browse, nor indeed foraging for the appropriate opportunity to browse. Evidently, whilst foraging and feeding are intrinsically linked, they are functionally quite different; foraging represents the appetitive phase of the process of acquiring nutrients which, for wide ranging herd dwelling social species such as Asian elephants will involve social interaction, information gathering and processing, collective and individual decision making and walking/locomotion. In contrast, feeding, the consummatory phase of acquiring nutrients in the form of grazing and browsing, likely involves significantly less cognition but more immediate and direct survival impacts. The AWPIS^©^ scores established here should be considered to represent the inherent value to the animal of both the appetitive and consummatory phases of the behaviour or cognitive process since from a welfare perspective, they are inextricably linked. So, satisfying the goal of highly scoring behaviours or cognitive process, cannot be assumed to fully address the psychological needs associated with them. Thus, it should be considered preferable, particularly for high AWPIS^©^ scoring behaviours and cognitive processes that animals are empowered to satisfy those goals themselves by being able to express appropriate motivated behaviours and cognitive processes, rather than have them reached for them by the provisioning of care by humans, see [15,32]. 

Whilst both the AZA and the British and Irish Association of Zoos and Aquariums (BIAZA) guidelines state browse material must be provided and also highlight the importance of foraging to elephants in captivity, neither mandate specific requirements in relations to time spent foraging, or opportunities to express grazing or browsing behaviours [24,25]. Such an omission should be addressed if these guidelines are truly configured to optimise captive elephant welfare. 

Walking was identified as the third most important priority with a consolidated AWPIS^©^ score of 65.5. As previously referred to, for both wild and captive elephants, a significant proportion of the time spent walking will overlap with foraging as well as other appetitive behaviours such as seeking social contact or shelter, and as such, it is unsurprising that walking has scored highly. Holdgate et al. [7] found that amongst 56 female elephants, of which 23 were Asian, distance walked increased with the diversity and unpredictability of feeding regimes but they found no association between distance walked and a variety of other behavioural, spatial and health related outcomes. The fact that unpredictable feeding regimes appear to have a greater influence on distance walked than space provided in captive environments [7] supports the consolidated AWPIS^©^ assessment that foraging is of psychological significance to Asian elephants and that walking may be a largely appetitive behaviour, frequently synonymous with foraging. As with other species, walking in Asian elephants is not a behaviour that in nature will be expressed for its own sake, rather it is expressed as a means to secure resources whether they be nutritional, social or physical, and as such, walking is largely appetitive in nature. However, given the centrality of walking to secure such evolutionary significant resources, it is likely that walking has some intrinsic value both physically and psychologically, a position supported by the work of Lewis et al. [35] who found that exercise decreased the incidence of foot pathologies in captive elephants and Morfield et al. [36] who demonstrated a link between staff-directed walking and a reduced risk of obesity in captive elephants.

Whilst bathing ranked low as a psychological priority according to both in and ex-situ assessments with a consolidated AWPIS^©^ score of 33.6, both AZA and BIAZA guidelines report benefits from pool use and subsequently mandate their availability [24,25]. It is possible that the discrepancy between the results of the AWPIS^©^ assessment and existing husbandry guidelines originate from an increased frequency with which captive elephants encounter bathing opportunities compared to their wild counterparts. This might further be compounded by the greater risks associated with bathing in the wild and the greater demands on the time of wild elephants to pursue behaviours that have more immediate impacts upon survival and reproduction. In comparison, the time required to sustain the physical needs of Asian elephants in captivity is compressed by the human provisioning of food, water and shelter, resulting in increased opportunities to interact with standing water more readily available in many captive elephant habitats. The perceived discrepancy in the hydrophily between wild and captive elephants, if it is accurate, may also reflect the differing thermoregulatory challenges of wild and captive environments, or even the differing social contexts with wild elephants often noted to convene socially around water sources. Furthermore, the extent to which captivity replicates the psychological priorities or evolutionary expectations of Asian elephants may also explain the perceived differences in the importance of bathing between wild and captive Asian elephants. Thus, captive elephants may actually utilise the pools as alternative outlets for other potentially frustrated motivations, and bathing may or may not represent perfectly acceptable alternative outlets for those frustrated motivations. Veasey et al. [52,53] challenged the presumption that differences in behaviour between wild and captive animals inevitably represent evidence of impoverished welfare, and in this case, it is possible that the anecdotally reported hydrophily of captive Asian elephants, see [24,25], if correct, may actually represent a positive welfare outcome of the captive state. However, the implications of these perceived differences would need to be assessed before forming any definitive conclusions on their actual welfare significance [52,53].

Regardless, the relatively low consolidated AWPIS^©^ score for bathing for Asian elephants should not necessarily lead to the conclusion that bathing opportunities should not be provisioned for. Rather, the value of bathing to captive elephant welfare should be considered in a pragmatic net welfare benefit context. Thus, given inevitable resource constraints, the prioritisation of finite resources to successfully secure foraging, browsing and grazing opportunities, which scored far higher than bathing, will likely yield greater welfare outcomes for captive elephants than providing costly filtered pools. This is particularly true in regions where pool use will be seasonally limited for climatic reasons. However, less expensive alternatives to permanent filtered pools which are of limited use and potentially hazardous during cooler seasons in temperate climates, should be considered such as seasonal wallows, pools or spray systems.

Another important insight from this assessment relates to the welfare significance of cognitive processes and social opportunities for Asian elephants. Historically, captive elephant management in zoos has emphasised maintaining physical health and a growing emphasis in the management of behaviour through behavioural enrichment and training. Whilst cognition may have been recognised as important, identifying the value of specific cognitive processes such as decision making, choice, learning, socialising, etc. that may have a value independent from their associated behaviours has long proven to be a challenge, see [18]. The process described here clearly identifies foraging and socialising as amongst the most important welfare priorities for the species, both of which have significant cognitive elements to them, underlining the importance cognition as well as behavioural and physical considerations in optimising welfare. 

The importance of sociality as is identified here appears to be corroborated by the work of Greco et al. [14] who identified five social variables that predicted stereotypic behaviour in elephants. Additionally, Meehan et al. [2] found that social variables were important predictors of behavioural indicators of elephant welfare, as well as endocrine dysfunction—a potentially stress-induced condition. That sociality emerged as the amongst the highest scoring priorities after those behaviours and cognitive processes essential for survival, combined with the evident connection between sociality and welfare outlined by Meehan et al. and Greco et al., further supports the recommendation that social factors should be prioritised in safeguarding the welfare of captive Asian elephants [2,4,12,14].

Beyond the discussion points specific to the management of Asian elephants in captivity, this pilot assessment illustrates the potential widespread utility of such a process in captive animal management and habitat design [37]. In managing animals in captivity, there is an inevitable tension between providing animals freedom to express normal (natural) behaviours whilst retaining sufficient control to protect them from pain, injury and disease, hunger and thirst, discomfort, fear and distress [15]. AWPIS^©^ not only provides a structure to better understanding those aspects of life in the wild with the greatest influence on welfare, but also provides a mechanism whereby psychological priorities can be more effectively considered within the context of safeguarding the physical needs of animals. 

In addition to helping navigate the tension between the physical and psychological needs of animals in captivity to optimise welfare, output from AWPIS^©^ assessments can also help optimise welfare in respect of the inevitable financial and spatial constraints of the captive environment. Based on the output generated here for example, captive elephants would most likely benefit from habitats that are of a size capable of sustaining natural feeding behaviours such as browsing, grazing and foraging for species-appropriate social units. Insights such as this can be used to prioritise resources where they will have the greatest positive impacts upon welfare, and also to determine whether or not it is possible to provision for a species’ psychological needs in a given environment. The evidence described here would suggest for example that zoos with limited space will face challenges in the provisioning of important behaviours related to foraging, feeding and sociality, whereas elephants with access to larger habitats capable of sustaining sources of appropriate vegetation for larger groups of elephants might not. It does not necessarily follow, however, that smaller, urban zoos should cease holding elephants, particularly given that walking does not appear to correlate with habitat size [7]. Rather, it confirms the necessity to provision for these priorities, and that solutions to these challenges will likely differ markedly in urban zoos in comparison to elephants in more extensive vegetated habitats where sustainable grazing may be realistically attainable. 

The AWPIS^©^ assessment described here has been configured to identify what is likely to be important to a species based on expert evaluations of behavioural and cognitive processes referencing wild populations, supported by insights from captive settings. AWPIS^©^ does not rely exclusively on elusive proofs of both negative and positive welfare impacts of each behaviour and cognitive process for the species being evaluated, but instead uses that evidence where it is available [37]. As it is currently configured AWPIS^©^ does not set out to recognize the value of behaviours expressed in captivity that would not regularly feature in the wild, nor does it attempt to recognise the value of behaviours and cognitive processes unique to domesticated species in captive scenarios. However, the higher scores provided by the ex-situ panel for processing food and problem solving in comparison to the in-situ panel, suggests that the methodology is sensitive to behaviours and cognitive processes expressed differentially in captive situations in comparison to the natural state, and therefore could be successfully applied to domesticated species.

A number of clear benefits of the AWPIS^©^ process are apparent over existing frameworks developed to help understand welfare and crucially to guide welfare management. It is a practical alternative to relying exclusively on extensive and often inconclusive epidemiological studies, it facilitates more effective and practical welfare prioritisation than other frameworks and better addresses the provisioning of positive emotional states. The process described here is also less constrained by existing management traditions than epidemiological research as it attempts to determine what is important to a species rather than what is the best management or housing systems of those currently utilised or under review. Subsequently, it is likely to be far more effective at delivering improvements in welfare than the more iterative paradigm that has prevailed in recent times based on reviews of existing systems [37]. Preference tests and consumer demand theory have also been used to help understand the psychological significance of discreet aspects of an animal’s existence [55,56], however, such experimental methodologies are not readily practicable in reviewing all behaviours and cognitive processes of a species, particularly for those species held outside research facilities. Furthermore, whilst the principle goal of the framework outlined here is not to assess welfare *per se*, output from the AWPIS^©^ process does have excellent potential to be used in welfare assessment; the extent to which facilities and management strategies provide for the priorities of species identified using the AWPIS^©^ tool, the more likely they are to cater for the species’ psychological needs and the higher welfare animals are likely to experience. 

Although the process requires convening a broad-based expert panel and is sufficiently complex to require a trained facilitator to supervise assessments, once a species-specific assessment has been completed, it benefits from being universally applicable to that species in any facility. A follow up assessment of the welfare priorities of Asian elephants might be improved by increasing the granularity of the behavioural and cognitive processes assessed; in particular, feeding could be further broken down by separating grazing and browsing, and similarly social interactions could be further subdivided. However, as has previously been alluded to in relation to walking and foraging, adding to the granularity of the ethogram and cognitive processes considered would also increase the overlap between behaviours and cognitive processes being assessed. A single assessment with a balanced panel would also be preferable to the consolidation of two independent assessments as it is likely that the collective nature of the process would decrease discrepancies between panellists with different backgrounds. Despite these issues, this and other pilot assessments [37] demonstrate that with consistent and facilitation, the AWPIS^©^ tool is a systematic, repeatable and defendable process to identify welfare priorities for captive care. So, in the hands of appropriately selected, informed, prepared and managed expert panels, AWPIS^©^ provides a framework for reasoned welfare prioritization in the absence of exhaustive experimental or epidemiological studies that is of genuine utility in guiding and assessing animal welfare [37]. 

## 5. Conclusions

It is evident that the AWPIS^©^ methodology described here represents a powerful tool in identifying welfare priorities. It is consistent in its output, provides an unparalleled tool to guide management to deliver optimal animal welfare and unique reference material by which welfare can be assessed. Here, it is shown that behaviours associated with immediate survival in the wild represent the greatest priorities for captive Asian elephant welfare closely followed by behaviours linked to sociality. The importance of appetitive behaviours and cognitive processes to the welfare of captive Asian elephants is also demonstrated. The process also demonstrates that challenges in securing elephant welfare will be context specific, and that the current understanding of the needs of Asian elephants in captivity, as manifest in existing husbandry guidelines, likely needs reconsidering.
